# Natural Polyphenols Versus Standard Therapy: Effects on Neuroinflammation and Alpha-Synuclein Expression in a 1-Methyl-4-Phenyl-1,2,3,6-Tetrahydropyridine (MPTP)-Induced Mouse Model of Parkinson’s Disease

**DOI:** 10.7759/cureus.103681

**Published:** 2026-02-15

**Authors:** Shalini Singh, Karan Suneja, Ipshita Jain, Suyog Sindhu, Satyendra Singh, Rishi Pal, Rakesh Dixit, Rajendra Nath

**Affiliations:** 1 Pharmacology and Therapeutics, King George's Medical University, Lucknow, IND; 2 Center for Advanced Research, King George's Medical University, Lucknow, IND

**Keywords:** neuroprotection, parkinson disease, polyphenols, tumor necrosis factor-alpha, α-synuclein

## Abstract

Background

Parkinson’s disease (PD) is a progressive neurodegenerative disorder characterized by dopaminergic neuronal loss, α-synuclein aggregation, and chronic neuroinflammation. While levodopa (L-DOPA) + carbidopa remains the cornerstone of symptomatic management, long-term use is associated with motor complications, prompting interest in adjunctive therapies targeting non-dopaminergic mechanisms. Polyphenolic compounds such as resveratrol and curcumin have demonstrated neuroprotective potential through antioxidant and anti-inflammatory actions. This study aimed to comparatively evaluate the effects of L-DOPA + carbidopa, resveratrol, curcumin, and their combination, resveratrol + curcumin, on motor function and biochemical markers in a 1-methyl-4-phenyl-1,2,3,6-tetrahydropyridine (MPTP)-induced mouse model of PD.

Methodology

An experimental study was conducted using 36 adult female C57BL/6 mice randomly allocated into six groups (*n* = 6): normal control group, MPTP group, L-DOPA + carbidopa group, resveratrol group, curcumin group, and resveratrol + curcumin group. PD was induced using intraperitoneal injection of MPTP (30 mg/kg/day for five days). Treatments were administered orally for 30 days. Motor coordination and locomotor activity were assessed using the rotarod test and open field tests at baseline (Day 0), post-MPTP induction (Day 6), and at the end of treatment (Day 37). Biochemical analysis of brain homogenates was performed to estimate α-synuclein and tumor necrosis factor-alpha (TNF-α) levels using enzyme-linked immunosorbent assay (ELISA). Statistical analysis was conducted using one-way analysis of variance (ANOVA) followed by Tukey’s post hoc test.

Results

MPTP administration resulted in significant motor impairment, elevated α-synuclein accumulation, and increased TNF-α levels compared with controls (*P* < 0.001). L-DOPA + carbidopa produced the greatest improvement in rotarod and open field performance and significantly reduced both α-synuclein and TNF-α levels. Resveratrol and curcumin monotherapy significantly improved motor performance and attenuated biochemical alterations compared with the MPTP group (*P* < 0.001 ), with resveratrol showing greater efficacy than curcumin. Combined treatment with resveratrol and curcumin resulted in superior motor recovery and greater reductions in α-synuclein and TNF-α levels than either compound alone.

Conclusions

The findings demonstrate a close association between motor dysfunction, α-synuclein accumulation, and neuroinflammation in MPTP-induced PD. L-DOPA+ carbidopa remains the most effective intervention for motor and biochemical parameters. However, resveratrol and curcumin, particularly in combination, exhibit significant neuroprotective effects and may serve as promising adjunctive strategies targeting disease-related inflammatory and synuclein-mediated pathways. These results support further investigation of polyphenolic compounds as complementary therapies in PD.

## Introduction

Parkinson's disease (PD) is a neurological illness that significantly impacts motor function, chronic and progressive in nature. Bradykinesia, muscle stiffness, resting tremor, and postural instability are among its primary motor symptoms. From a pathogenic perspective, PD is characterized by intraneuronal aggregation of misfolded α-synuclein into Lewy bodies and degeneration of dopaminergic neurons in the substantia nigra pars compacta [1,2]. While dopamine depletion accounts for many of the motor symptoms observed in PD, growing evidence indicates that multiple molecular and cellular mechanisms contribute to disease onset and progression [3]. However, the precise interplay among α-synuclein aggregation, neuroinflammatory activation, and dopaminergic neuronal loss remains incompletely understood, and their relative contributions to motor dysfunction continue to be debated, highlighting a critical translational gap. α-Synuclein plays a central role in PD pathogenesis. Aberrant aggregation of this presynaptic protein disrupts synaptic integrity and promotes neuronal injury. Recent research suggests that altered α-synuclein levels and its aggregated species may serve as valuable biomarkers for monitoring disease activity and therapeutic response in experimental PD models [2,4]. Notably, α-synuclein pathology is closely associated with neuroinflammatory pathways, which further exacerbate neuronal degeneration [3,5]. Neuroinflammation is now recognized as an active contributor to PD rather than merely a consequence of neuronal loss. Activated microglia release pro-inflammatory mediators that enhance oxidative stress and accelerate neurodegeneration. Among these mediators, tumor necrosis factor-alpha (TNF-α) has been consistently implicated in dopaminergic neuronal toxicity and motor dysfunction in animal models of PD [5-7]. Levodopa (L-DOPA), a direct metabolic precursor of dopamine, remains the cornerstone of PD pharmacotherapy. However, prolonged administration is associated with significant motor complications, including L-DOPA-induced dyskinesia (LID) [8]. These limitations have prompted increasing interest in therapeutic strategies targeting non-dopaminergic mechanisms such as oxidative stress and inflammation. In this context, naturally occurring polyphenolic compounds such as resveratrol and curcumin have demonstrated promising neuroprotective effects in preclinical studies, primarily through their antioxidant and anti-inflammatory properties [9,10]. The MPTP-induced mouse model is widely used in PD research as it replicates dopaminergic neuronal loss and motor impairments similar to those observed in human disease [11]. Behavioral assessments, including the rotarod and open-field tests, are routinely employed to evaluate motor coordination, balance, and spontaneous locomotor activity in this model [8,12]. When integrated with biochemical estimation of α-synuclein and TNF-α levels, these behavioral evaluations enable meaningful correlation between functional deficits and underlying molecular changes. The present study was conducted to evaluate the comparative effects of L-DOPA + carbidopa, resveratrol, and curcumin on motor behavior and biochemical markers in an MPTP-induced mouse model of PD.

## Materials and methods

Study setting

This experimental study was conducted at the Department of Pharmacology in collaboration with the Centre for Advanced Research (CFAR), King George's Medical University (KGMU), Lucknow, Uttar Pradesh, India. PD was induced in mice using a subacute dose of 1-methyl-4-phenyl-1,2,3,6-tetrahydropyridine (MPTP, Sigma-Aldrich, St. Louis, MO; catalog no. M0896). The Institutional Animal Ethics Committee (IAEC) at KGMU (project no. 198/IAEC/2024) evaluated and validated all experimental procedures, and the experiments were carried out in compliance with the relevant national guidelines for the use and care of laboratory animals. The study took four months to complete.

Experimental animals

A total of 36 healthy adult female C57BL/6 mice [13], weighing 16-20 g, were used in the study. Animals were procured from a CCSEA-certified breeding facility at the Central Drug Research Institute (CDRI), Lucknow, Uttar Pradesh, India. Mice were kept in the institutional animal house under monitored settings with a regulated temperature of 22 ± 2 °C and a 12-hour light-dark cycle (lights on from 8 am to 8 pm). Regular standard pellet diet and water were readily accessible. Before the experiment commenced, the animals were acclimated to the lab environment for a week. Animal health and welfare were regularly monitored by a veterinarian in accordance with CCSEA guidelines [14]. After acclimatization, mice were randomly divided into six groups consisting of six mice in each group [8].

PD mice model

MPTP was obtained in powder form and freshly dissolved in sterile normal saline before administration. Mice were given MPTP intraperitoneally once a day for five days in succession at a dose of 30 mg/kg to develop PD [15].

Reagents and drugs procurement

MPTP (catalog no. M0896) was purchased from Sigma-Aldrich. Resveratrol and curcumin were obtained from Tokyo Chemical Industry (TCI), Tokyo, Japan. L-DOPA + carbidopa was procured from Sun Pharmaceutical Industries Ltd., Mumbai, India.

Experimental groups and drug treatment

**Figure 1:**
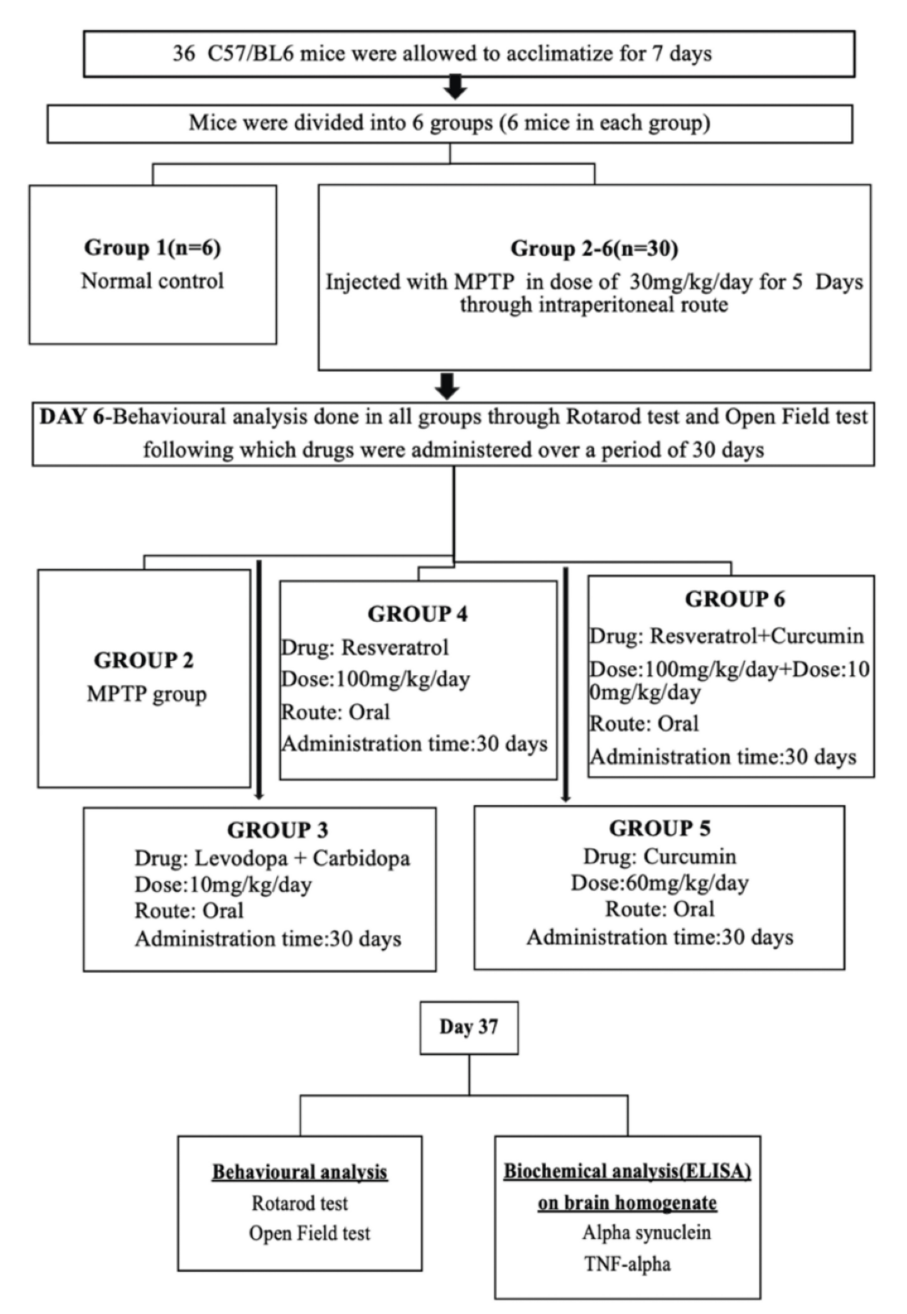
Research methodology. ELISA, enzyme-linked immunosorbent assay; TNF-α, tumor necrosis factor-alpha

As shown in Figure 1 of the research methodology, the mice were randomly distributed into six experimental groups (*n* = 6). Group 1 (Normal Control): Mice received a normal pellet diet and water ad libitum throughout the study period. Group 2 (PD Control-MPTP group): Mice were administered MPTP (30 mg/kg, intraperitoneal) for five consecutive days to induce PD. Group 3 (L-DOPA + Carbidopa Group): MPTP-induced mice received L-DOPA + carbidopa at a dose of 10 mg/kg/day orally for 30 days [8]. Group 4 (Resveratrol Group): MPTP-induced mice received resveratrol at a dose of 100 mg/kg/day orally for 30 days [9]. Group 5 (Curcumin Group): MPTP-induced mice received curcumin at a dose of 60 mg/kg/day orally for 30 days [10]. Group 6 (Resveratrol + Curcumin Group): MPTP-induced mice received a combination of resveratrol (100 mg/kg/day) and curcumin (60 mg/kg/day) orally for 30 days.

Parameters assessed

Behavioral Tests

Behavioral assessments were performed to evaluate motor coordination, locomotor activity, and exploratory behavior in mice. All behavioral tests were conducted during the light phase of the cycle in a quiet, temperature-controlled room. Animals were acclimatized to the testing environment before data collection, and the instrument was disinfected with 70% ethanol after every experiment session to get rid of odor indications.

Rotarod Test

Motor coordination and balance were assessed using the institutional rotarod apparatus (rod length: 75 cm; diameter: 30 mm). Animals were acclimatized to the behavioral testing room for at least one hour before experimentation to minimize stress-induced behavioral variability. During testing, each mouse was placed on an accelerating rotating rod, progressively increasing its speed from 4 to 40 rpm over 300 seconds. For each trial, the time needed to fall off the rotating rod was measured to assess motor coordination and balance. A maximum cutoff time of 300 seconds was applied to prevent fatigue and potential injury; animals remaining on the rod for the entire duration were assigned the cutoff value. Each mouse underwent three trials per test session, with a 15-minute rest interval between consecutive trials to allow recovery and minimize fatigue and carry-over effects. If a mouse passively rotated by clinging to the rod for two consecutive rotations without active walking, the trial was terminated. The average latency to fall across the three trials was calculated for each animal and used for subsequent statistical analysis.

Open Field Test

The open field activity test was used to measure spontaneous locomotor activity and exploratory behavior. The institutional apparatus consisted of a square arena (76 × 76 × 50 cm) with a white floor and was enclosed by privacy shades to eliminate any unwanted external visual cues. Animals were acclimatized to the testing room for at least one hour before experimentation to minimize stress-related behavioral variability. Each mice were placed in a corner of the arena and permitted to move about freely for 5 minutes. Their locomotor activity was constantly tracked using an overhead camera coupled to an automated video tracking system (AnyMaze® software, Stoelting Co., Wood Dale, IL). Parameters, including total distance travelled, number of squares crossed, average velocity, and rearing frequency, were automatically quantified and analyzed.

Biochemical tests

Biochemical tests were conducted to assess molecular changes associated with PD. Levels of α-synuclein and TNF-α in brain tissue were measured using enzyme-linked immunosorbent assay (ELISA). These markers reflect protein aggregation and neuroinflammation, respectively.

Alpha-Synuclein and TNF-α

In the last phase of the experiment, mice were euthanized using a high dose of sodium phenobarbitone (200 mg/kg, intraperitoneal). The brains were immediately excised, rinsed with ice-cold phosphate-buffered saline (PBS), and biochemically analyzed. Brain tissues were homogenized in cold PBS using a mechanical homogenizer, and the homogenates were centrifuged to remove cellular debris. The clear supernatant (brain homogenate) was collected and preserved at -20 °C until subsequent analysis. Levels of α-synuclein and TNF-α in brain homogenates were quantified using ELISA. α-Synuclein was estimated using a Mouse SNCA ELISA kit (FineTest, Wuhan Fine Biotech Co., Ltd., Wuhan; Code: EM1369), while TNF-α levels were measured using a Mouse TNF-α ELISA kit (FineTest; Code: EM0183). Absorbance was measured with a microplate reader at the specified wavelength, and concentrations were determined using the standard curves included with the kits.

Statistical analysis

Statistical analysis was performed using the institutionally licensed SPSS software, version 24.0 (IBM Corp., Armonk, NY). Data were analyzed using one-way analysis of variance (ANOVA), followed by Tukey’s post hoc test for multiple group comparisons. Results are expressed as mean ± standard deviation (SD). A *P*-value < 0.05 was considered statistically significant.

## Results

Behavioral parameters assessed by the rotarod

Rotarod assessment demonstrated comparable motor coordination among all experimental groups at baseline (Day 0), with no statistically significant inter-group differences in latency to fall (*P* = 0.83), indicating uniform baseline motor performance as shown in Table 1. Following MPTP administration (Day 6), a marked and significant reduction in rotarod latency was observed in all MPTP-induced PD groups compared with the control group (*P* < 0.0001), confirming successful induction of PD. Post-hoc Tukey analysis revealed no significant differences among the MPTP-induced groups at this time point (*P *> 0.05), indicating comparable disease severity before initiation of treatment. At the end of the treatment (Day 37), one-way ANOVA revealed a highly significant difference in rotarod performance among the groups (*P *< 0.0001). The MPTP group continued to exhibit severe motor deficits compared with the normal control group (*P* < 0.001). Treatment with L-DOPA + carbidopa resulted in the greatest improvement in latency to fall and was significantly superior to the MPTP group (*P* < 0.001). Combined treatment with resveratrol + curcumin produced a significant improvement compared with the MPTP group (*P* < 0.001) but was inferior to L-DOPA + carbidopa (*P* < 0.01). Resveratrol monotherapy showed moderate recovery and was significantly better than the MPTP group (*P* < 0.001) but inferior to combination therapy (*P* < 0.01). Curcumin monotherapy demonstrated the least improvement among the treatment groups, though performance remained significantly better than the MPTP group (*P* < 0.001).

**Table 1:** Effect of levodopa + carbidopa, resveratrol, and curcumin on the rotarod test in MPTP-induced Parkinson’s disease mice. Rotarod latency to fall (seconds) measured with a cutoff time of 300 s. Values are expressed as mean ± standard deviation (SD) derived from the average of three trials per mouse (*n* = 6 mice per group). Measurements were recorded at baseline (Day 0), after MPTP administration and before treatment initiation (Day 6), and at the end of the treatment period (Day 37). A *P*-value < 0.05 was considered statistically significant. MPTP, 1-methyl-4-phenyl-1,2,3,6-tetrahydropyridine; ANOVA, analysis of variance

Group	Day 0	Day 6	Day 37
Normal Control	291.2 ± 3.0	289.5 ± 2.9	292.2 ± 3.0
MPTP	290.3 ± 2.8	94.5 ± 2.6	76.7 ± 2.5
Levodopa + Carbidopa	291.0 ± 2.7	94.5 ± 2.0	243.0 ± 7.0
Resveratrol	290.8 ± 2.8	93.8 ± 2.0	179.2 ± 5.0
Curcumin	289.5 ± 2.7	92.8 ± 2.0	150.3 ± 4.0
Resveratrol + Curcumin	292.0 ± 2.9	94.3 ± 2.0	210.7 ± 7.5
One-way ANOVA	*F *= 0.41, *P* = 0.83	*F *= 948.6, *P* =< 0.0001	*F *= 612.4, *P*=< 0.0001

Behavioral parameters assessed by the open field test

Open field assessment demonstrated comparable locomotor and exploratory behavior across all experimental groups at baseline (Day 0), with no statistically significant inter-group differences in total distance travelled, average velocity, number of squares crossed, or rearing frequency (*P* > 0.05 for all parameters), indicating uniform baseline motor and exploratory performance, as shown in Table 2. Following MPTP administration (Day 6), all MPTP-induced PD groups exhibited a marked and significant reduction in locomotion and exploratory activity compared with the control group, as evidenced by significant decreases in distance travelled, velocity, squares crossed, and rearing frequency (*P* < 0.0001 for all parameters). Tukey’s post hoc analysis revealed no significant differences among the MPTP-induced groups at this time point (*P* > 0.05), confirming comparable disease severity before initiation of treatment. At the end of the treatment period (Day 37), one-way ANOVA revealed highly significant differences among groups for all open field parameters (*P* < 0.0001). The MPTP group continued to show persistent reduced locomotor and exploratory behavior compared with the control group (*P* < 0.001). Treatment with L-DOPA + carbidopa resulted in the greatest improvement across all parameters, with significant distance increases travelled, velocity, squares crossed, and rearing frequency compared with the MPTP group (*P* < 0.001). Combined treatment with resveratrol + curcumin produced significant improvement in all parameters compared with the MPTP group (*P* < 0.001) but remained inferior to L-DOPA + carbidopa (*P* < 0.01). Resveratrol monotherapy demonstrated moderate recovery and was significantly superior to curcumin monotherapy (*P* < 0.05), while both treatments were significantly better than the MPTP group (*P* < 0.001). 

**Table 2:** Effect of levodopa + carbidopa, resveratrol, curcumin, and resveratrol + curcumin on spontaneous locomotor and exploratory behavior assessed using the open field test in MPTP-induced Parkinson’s disease mice. Total distance travelled, number of squares crossed, average velocity, and rearing frequency were recorded at baseline (Day 0), at the start of treatment following MPTP administration (Day 6), and at the end of treatment (Day 37). Data are expressed as mean ± standard deviation (SD). A *P*-value < 0.05 was considered statistically significant. MPTP, 1-methyl-4-phenyl-1,2,3,6-tetrahydropyridine; ANOVA, analysis of variance

Day	Group	Distance travelled (cm)	Velocity (cm/s)	Squares crossed	Rearing frequency
Day 0	Normal Control	1185 ± 30	3.95 ± 0.09	93.2 ± 2.4	23.6 ± 1.9
	MPTP	1178 ± 32	3.93 ± 0.10	92.8 ± 2.7	23.1 ± 2.1
	Levodopa + Carbidopa	1186 ± 28	3.96 ± 0.08	93.4 ± 2.2	23.4 ± 1.8
	Resveratrol	1181 ± 29	3.94 ± 0.09	92.9 ± 2.3	23.0 ± 2.0
	Curcumin	1179 ± 31	3.93 ± 0.10	92.6 ± 2.5	22.8 ± 2.2
	Resveratrol + Curcumin	1184 ± 27	3.95 ± 0.08	93.1 ± 2.1	23.3 ± 1.7
	ANOVA	*F* = 0.42, *P* > 0.70	*F* = 0.38, *P* > 0.75	*F* = 0.36, *P* > 0.80	*F* = 0.44, *P* > 0.70
Day 6	Normal Control	1172 ± 24	3.90 ± 0.08	92.5 ± 2.2	23.3 ± 1.8
	MPTP	380 ± 60	1.25 ± 0.18	32.4 ± 5.8	8.6 ± 1.5
	Levodopa + Carbidopa	400 ± 65	1.32 ± 0.20	34.2 ± 6.2	9.2 ± 1.7
	Resveratrol	395 ± 58	1.30 ± 0.17	33.6 ± 5.5	8.9 ± 1.6
	Curcumin	360 ± 55	1.18 ± 0.16	30.8 ± 5.1	7.9 ± 1.4
	Resveratrol + Curcumin	420 ± 70	1.38 ± 0.21	36.1 ± 6.5	9.8 ± 1.8
	ANOVA	*F* = 150, *P* < 0.0001	*F* = 130, *P* < 0.0001	*F* = 165, *P* < 0.0001	*F* = 110, *P* < 0.0001
Day 37	Normal Control	1193 ± 26	3.98 ± 0.09	96.4 ± 2.3	24.0 ± 1.8
	MPTP	700 ± 90	2.30 ± 0.28	55.2 ± 7.4	12.3 ± 2.1
	Levodopa + Carbidopa	1080 ± 95	3.60 ± 0.30	91.5 ± 5.2	20.4 ± 2.5
	Resveratrol	820 ± 85	2.75 ± 0.27	68.0 ± 6.5	15.3 ± 2.2
	Curcumin	760 ± 80	2.55 ± 0.25	61.2 ± 6.0	13.9 ± 2.0
	Resveratrol + Curcumin	950 ± 92	3.15 ± 0.29	78.4 ± 7.2	17.8 ± 2.3
	ANOVA	*F* = 78, *P* < 0.0001	*F* = 72, *P* < 0.0001	*F* = 85, *P* < 0.0001	*F* = 58, *P* < 0.0001

Biochemical parameter assessed by α-synuclein levels

**Figure 2:**
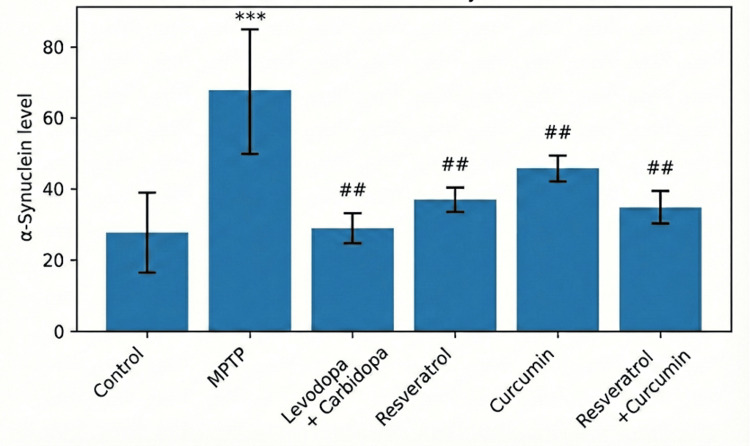
Effect of treatments on α-synuclein levels. The figure depicts the group-wise distribution of α-synuclein levels expressed as mean ± SD. The MPTP group shows a pronounced elevation in α-synuclein compared to the normal control group (***P < 0.001), whereas all treatment groups demonstrate significant reductions compared to the MPTP group (##*P* < 0.001). The graphical representation depicts that levodopa + carbidopa produced the greatest reduction, followed by the resveratrol+ curcumin combination, which was more effective than either compound alone. MPTP, 1-methyl-4-phenyl-1,2,3,6-tetrahydropyridine; SD, standard deviation

Quantitative estimation of α-synuclein levels in brain homogenates demonstrated significant intergroup differences, as shown in Table 3. Figure 2 depicts the normal control group showing a physiological level of α-synuclein expression, representing normal protein levels. In contrast, MPTP administration produced a substantial elevation in α-synuclein levels compared with the normal control group (*P* < 0.001), confirming successful induction of synucleinopathy associated with dopaminergic neurotoxicity. Treatment with L-DOPA + carbidopa resulted in a notable reduction in α-synuclein levels compared with the MPTP group (*P* < 0.001), with values comparable to those of the normal control group (*P* > 0.05). Resveratrol treatment also led to a decrease in α-synuclein levels relative to the MPTP group (*P* < 0.01), although levels remained modestly higher than control (*P* < 0.05). Curcumin administration produced a significant attenuation of α-synuclein expression compared with the MPTP group (*P* < 0.05) but was less effective than L-DOPA + carbidopa and resveratrol. Notably, combined treatment with resveratrol and curcumin resulted in a greater reduction in α-synuclein levels compared with the MPTP group (*P* < 0.001) and showed numerically lower levels than either compound administered alone; however, inter-treatment comparisons among L-DOPA + carbidopa, resveratrol, curcumin, and resveratrol + curcumin did not reach statistical significance (*P* > 0.05)

**Table 3:** Effect of levodopa + carbidopa, resveratrol, curcumin, and resveratrol + curcumin on α-synuclein levels in brain homogenates of MPTP-induced Parkinson’s disease mice. Data are presented as mean ± standard deviation (SD). A *P*-value < 0.05 was considered statistically significant. MPTP, 1-methyl-4-phenyl-1,2,3,6-tetrahydropyridine; ANOVA, analysis of variance

Group	α-Synuclein (Mean ± SD)
Normal Control	27.60 ± 11.15
MPTP	67.14 ± 21.35
Levodopa + Carbidopa	28.74 ± 4.16
Resveratrol	36.77 ± 3.46
Curcumin	45.51 ± 3.68
Resveratrol + Curcumin	34.68 ± 4.56
One-way ANOVA	*F* = 18.62, *P* < 0.001

Biochemical parameter assessed by TNF-α levels

**Figure 3:**
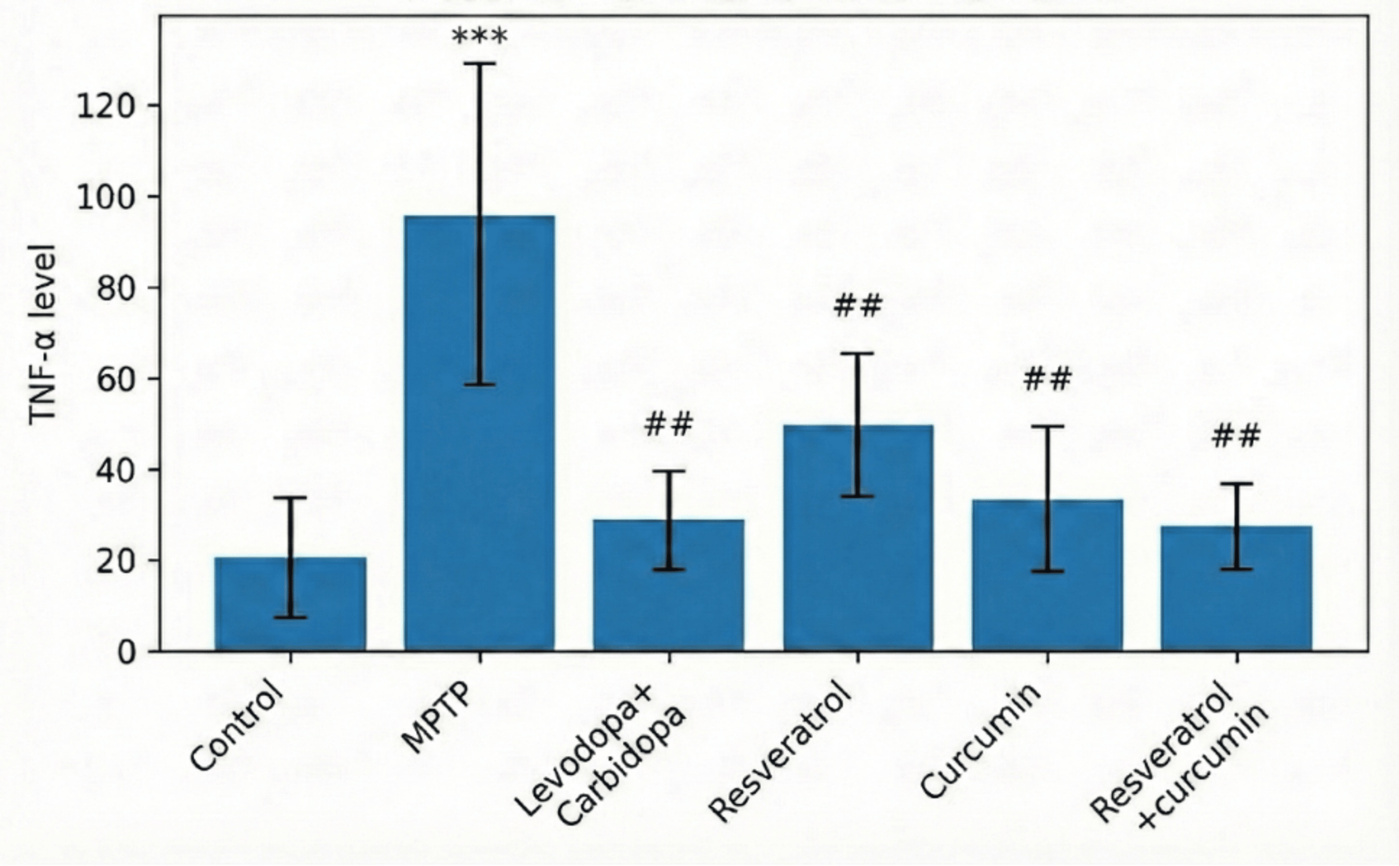
Effect of treatments on TNF-α levels. Depicts TNF-α levels across experimental groups. A significant elevation is observed in the MPTP group compared to the control (***P < 0.001). Treatment with levodopa + carbidopa, resveratrol, curcumin, and resveratrol + curcumin significantly reduced TNF-α levels compared to the MPTP group (##*P* < 0.01). The graphical representation depicts that levodopa + carbidopa produced the greatest reduction, followed by the resveratrol + curcumin combination, which was more effective than either compound alone. MPTP, 1-methyl-4-phenyl-1,2,3,6-tetrahydropyridine; TNF-α, tumor necrosis factor-α

Analysis of TNF-α levels revealed marked neuroinflammatory alterations across the experimental groups, as shown in Table 4. Figure 3 depicts the normal control group exhibiting low basal TNF-α expression, whereas MPTP administration resulted in a significant elevation in TNF-α levels compared with the normal control group (*P* < 0.001), indicating activation of inflammatory pathways following dopaminergic neuronal injury. Treatment with L-DOPA + carbidopa produced a significant reduction in TNF-α levels compared with the MPTP group (*P* < 0.001), restoring values comparable to those of the normal control group (*P* > 0.05). Curcumin administration led to a significant attenuation of TNF-α expression compared with the MPTP group (*P* < 0.01), suggesting a robust anti-inflammatory effect. Resveratrol treatment also resulted in a decrease in TNF-α levels relative to the MPTP group (*P* < 0.01), although the magnitude of reduction was comparatively moderate. Importantly, combined treatment with resveratrol + curcumin produced a marked reduction in TNF-α levels compared with the MPTP group (*P* < 0.001) and demonstrated levels comparable to those observed with L-DOPA + carbidopa and the normal control group (*P* > 0.05). One-way ANOVA confirmed a highly significant overall difference among groups (*P* < 0.001), and Tukey’s post hoc analysis verified that the MPTP group differed significantly from all treatment groups (*P* < 0.05), while no significant differences were observed between the control and treated groups (*P* > 0.05).

**Table 4:** Effect of levodopa + carbidopa, resveratrol, curcumin, and resveratrol + curcumin on tumor necrosis factor-α (TNF-α) levels in brain homogenates of MPTP-induced Parkinson’s disease mice. Data are expressed as mean ± standard deviation (SD). A *P*-value < 0.05 was considered statistically significant. MPTP, 1-methyl-4-phenyl-1,2,3,6-tetrahydropyridine; ANOVA, analysis of variance

Group	TNF-α (Mean ± SD)
Normal Control	20.58 ± 13.13
MPTP	95.78 ± 37.27
Levodopa + Carbidopa	28.72 ± 10.80
Resveratrol	49.64 ± 15.79
Curcumin	33.42 ± 15.89
Resveratrol + Curcumin	27.43 ± 9.43
One-way ANOVA	*F* = 14.87, *P* < 0.001

## Discussion

This study shows that MPTP administration successfully induced Parkinsonian features in C57BL/6 mice. This was evidenced by significant motor impairment, increased accumulation of α-synuclein, and elevated TNF-α levels in brain homogenates. These findings confirm effective dopaminergic neurotoxicity and synucleinopathy, which are key pathological characteristics of PD [1,16]. The motor deficits observed in the rotarod and open-field tests closely matched the biochemical changes, supporting the value of combining behavioral and molecular assessments in experimental PD studies. Treatment with L-DOPA + carbidopa produced the greatest improvement in motor coordination and locomotor activity, along with marked reductions in α-synuclein and TNF-α levels. This strong effect is consistent with L-DOPA + carbidopa's established role as the gold-standard therapy for PD, primarily through restoration of dopamine levels in the nigrostriatal pathway [15,17]. These findings highlight the close relationship between motor improvement and stabilization of inflammatory and synaptic processes in the Parkinsonian brain. Resveratrol treatment led to significant but moderate improvements in motor behavior and biochemical parameters compared to L-DOPA + carbidopa. These results agree with previous experimental studies demonstrating resveratrol’s neuroprotective effects in PD models, largely due to its antioxidant and anti-inflammatory properties [18]. Resveratrol has been shown to reduce microglial activation, suppress pro-inflammatory cytokine release, and decrease α-synuclein expression. These findings imply that while resveratrol confers neuroprotective benefits, its effects are more consistent with an adjunct or disease-modifying role than with primary symptomatic management. Curcumin administration also resulted in measurable improvements in motor performance and inflammatory status, although the effects were less pronounced than those seen with resveratrol or L-DOPA + carbidopa. Curcumin is known to exert neuroprotective effects by inhibiting TNF-α-mediated inflammation and reducing oxidative stress in MPTP-induced PD models [10,19]. The reduction in TNF-α levels observed in the curcumin-treated group further supports the important role of inflammatory mechanisms in PD progression, despite curcumin’s known limitations related to bioavailability. Importantly, combined treatment with resveratrol + curcumin produced greater improvements in motor function and more substantial reductions in α-synuclein and TNF-α levels compared with either compound alone. This effect suggests enhanced benefit between the two polyphenols, likely due to their complementary actions on oxidative stress, neuroinflammation, and protein aggregation [18]. Overall, the close association between behavioral recovery and attenuation of α-synuclein and TNF-α levels across treatment groups indicates a strong link between motor function and underlying molecular pathology. Since α-synuclein aggregation and chronic neuroinflammation are central drivers of PD progression, their reduction is considered essential for meaningful neuroprotection [20]. Although the study is limited by its short duration and use of a single toxin-based model, the combined behavioral and biochemical findings enhance its translational relevance. Further studies with longer treatment durations and additional molecular markers are warranted to better define the disease-modifying potential of these interventions.

## Conclusions

This study provides evidence that MPTP-induced motor deficits are strongly linked to increased α-synuclein aggregation and heightened neuroinflammatory activity, as indicated by elevated TNF-α levels. These results emphasize the interdependent roles of pathological protein accumulation, inflammation, and motor dysfunction in the progression of experimental PD. L-DOPA + carbidopa produced the most robust and consistent restoration of motor performance and biochemical balance, reaffirming its central role as the cornerstone of PD therapy. Importantly, both resveratrol and curcumin demonstrated meaningful neuroprotective effects, reflected by reductions in α-synuclein levels and suppression of inflammatory markers. Notably, combined administration of these polyphenolic agents yielded superior outcomes compared with monotherapy, suggesting additive benefits arising from their complementary actions on oxidative stress, inflammatory pathways, and synuclein-associated pathology. Together, these findings support the potential role of polyphenolic compounds as adjunctive neuroprotective strategies alongside standard dopaminergic treatment. Moreover, the study highlights the importance of concurrently addressing motor deficits and underlying molecular mechanisms to achieve more effective and comprehensive neuroprotection in experimental models of PD.
